# End-to-end 3D instance segmentation of synthetic data and embryo microscopy images with a 3D Mask R-CNN

**DOI:** 10.3389/fbinf.2024.1497539

**Published:** 2025-01-29

**Authors:** Gabriel David, Emmanuel Faure

**Affiliations:** Laboratoire d’Informatique, de Robotique et de Micro-électronique de Montpellier, Centre National de la Recherche Scientifique, Université Montpellier, Montpellier, France

**Keywords:** 3D deep learning, instance segmentation, Mask R-CNN, microscopy, *Phallusia mammillata*, embryos, synthetic dataset, tensorflow

## Abstract

In recent years, the exploitation of three-dimensional (3D) data in deep learning has gained momentum despite its inherent challenges. The necessity of 3D approaches arises from the limitations of two-dimensional (2D) techniques when applied to 3D data due to the lack of global context. A critical task in medical and microscopy 3D image analysis is instance segmentation, which is inherently complex due to the need for accurately identifying and segmenting multiple object instances in an image. Here, we introduce a 3D adaptation of the Mask R-CNN, a powerful end-to-end network designed for instance segmentation. Our implementation adapts a widely used 2D TensorFlow Mask R-CNN by developing custom TensorFlow operations for 3D Non-Max Suppression and 3D Crop And Resize, facilitating efficient training and inference on 3D data. We validate our 3D Mask R-CNN on two experiences. The first experience uses a controlled environment of synthetic data with instances exhibiting a wide range of anisotropy and noise. Our model achieves good results while illustrating the limit of the 3D Mask R-CNN for the noisiest objects. Second, applying it to real-world data involving cell instance segmentation during the morphogenesis of the ascidian embryo *Phallusia mammillata*, we show that our 3D Mask R-CNN outperforms the state-of-the-art method, achieving high recall and precision scores. The model preserves cell connectivity, which is crucial for applications in quantitative study. Our implementation is open source, ensuring reproducibility and facilitating further research in 3D deep learning.

## 1 Introduction

In recent years, we have observed a growing interest in directly exploiting three-dimensional (3D) data in deep learning despite the challenges it presents (Yang et al., 2024). These challenges include the cost of data acquisition, annotation, and storage, as well as the need for substantial computing power for manipulating the volume of data. Many reasons concur with this development, such as the increasing availability of sufficiently voluminous 3D datasets for learning and the trivialization of adequate computing resources. However, the primary reason is that the real world evolves in three dimensions (Ahmed et al., 2018). Two-dimensional (2D) approaches applied to 3D data limit the learning performance because they lack the global context of the whole image. This is, in particular, the case for the voxel images that are used in medical or microscopy imaging, in which 3D deep learning has found numerous applications (Singh et al., 2020; Liu et al., 2021) to replace or complete 2D approaches (von Chamier et al., 2021).

One important but still challenging task that 3D deep learning offers to solve is instance segmentation in these types of images. Instance segmentation is already considered as the most complicated task in 2D deep learning (Hafiz and Bhat, 2020). Its difficulty is inherited from object detection (Girshick et al., 2014). Classical end-to-end supervised learning assumes there is a one-to-one mapping between the input and the ground truth output (Shalev-Shwartz and Ben-David, 2014). However, there exist M! manners to assign in parallel M labels to M object instances in an input image. The existence of a non-sequential supervised method to instantiate objects at one time is still an open question. The recent deep learning literature nonetheless proposes prominent sequential approaches to circumvent this problem. These approaches mainly involve predicting feature maps, such as instance contours or shape patches, using a deep learning approach of semantic segmentation, followed by a post-processing step to achieve instance segmentation (Wolny et al., 2020; Eschweiler et al., 2019; Hirsch et al., 2020). This post-processing is usually a watershed-like method preceded by more or less elaborated steps in order to optimize the label seed locations. The actual state-of-art approach for cell instance segmentation, Cellpose, addresses 3D instance segmentation by fusing the 2D predictions of its original method based on vector fields (Stringer et al., 2020; Pachitariu and Stringer, 2022; Stringer and Pachitariu, 2024). However, the literature still lacks a competitive end-to-end and generic approach that addresses the 3D instance segmentation problem.

Here, we introduce this missing piece, which is the 3D Mask R-CNN network. Mask R-CNN is a powerful region-based end-to-end network that detects and segments many classes, up to 80, in its 2D application on the COCO dataset (He et al., 2017; Lin et al., 2014). It relies on the region-based paradigm (Girshick, 2015; Ren et al., 2017), which consists of first individualizing the instance regions of interest (RoIs) and then segmenting the object within, in contrast to a segment-first method.

We base our implementation on a well-known and widely used two-dimensional TensorFlow implementation (Abdulla, 2017), and we adapt all its operations to process 3D input tensors. In particular, to benefit from TensorFlow core execution speed, we propose the 3D Non Max Suppression and 3D Crop And Resize algorithms as TensorFlow custom operations because native algorithms only apply to 2D images or features. This adaptation allows the delivery of a truly end-to-end implementation with acceptable training and inference time.

We validate our 3D Mask R-CNN with two experiences. The first one involves training the network within a controlled environment that consists of noisy images of objects that can belong to three classes: cuboid, ellipsoid, and irregular pyramid. The Mask R-CNN then gives good results in the evaluation. Finally, we apply our network in the real case of cell instance segmentation during the morphogenesis of the ascidian *Phallusia mammillata* (PM) (Guignard et al., 2020a). We show that, although this second case constitutes an extreme regime for the Mask R-CNN, our implementation appears competitive against the state-of-the-art method for 3D instance segmentation in bio-imaging and illustrates itself in feature conservation. We provide the code of our 3D Mask R-CNN on GitHub (David, 2024).

## 2 Methods

### 2.1 Network architecture

The 3D Mask R-CNN is composed of two successive blocks: an initial Region Proposal Network (RPN) and a second part, often called the Head. The RPN takes the whole image as input, while the Head works on the RoI proposals extracted from the RPN feature maps.

#### 2.1.1 Region proposal network

The goal of the RPN is to detect objects in the input image without regard to their specific classes. To achieve this, it uses a system of predefined RoI proposals, also called anchors (Lin et al., 2017). From these anchors projected over the input image, the RPN aims (i) to determine which anchors are positive (overlapping with an object in the input) or negative (not overlapping with ground truth instances) and (ii) to predict the adjustments needed for each positive anchor to best match the object bounding boxes. The RPN is composed of a classifier backbone, usually a Residual Network (He et al., 2016) with pyramidal features (Lin et al., 2017), and a special layer that maps each anchor to a vector. These vectors are used by a classifier and a regressor that fulfill the RPN purpose. Due to the large number of anchors, the RPN samples the most relevant anchors, applies the predicted adjustments, and sorts them using a Non Max Suppression (NMS) algorithm to obtain the RoI proposals. These proposals are cropped from the appropriate RPN feature maps and resized to predetermined shapes for the Head modules. The cropping step relies on the RoIAlign approach (He et al., 2017), which samples the feature maps in the normalized space in order to avoid the importance of the quantification effect caused by the classical RoIPooling method.

#### 2.1.2 Head

The Mask R-CNN Head is composed of three branches: the regression, classification, and segmentation modules. The classification and regression modules refine the class and the bounding box predictions from the RPN. Although the RPN only determines if an anchor contains an object regardless of its precise class, the Head classifier identifies the object class within the submitted RoI, including the background class. The Head regressor shares the RPN regressor’s objective of inferring the adjustment to apply to RoI proposals to match the instance. The segmentation module finally decodes the masks of the detected objects from the RoI maps. During training, these three modules are trained simultaneously. Meanwhile, in prediction, the RoI proposals are first submitted to the Head classifier and regressor. The RoIs are then modified and sorted according to the regressor and classifier predictions with an NMS method by class and finally submitted to the segmentation module. An instance segmentation image is thus obtainable at this point.

### 2.2 3D Non Max Suppression and crop and resize

As the Mask R-CNN uses the TensorFlow NMS and Crop And Resize (CAR) methods, and because we want to benefit from the TensorFlow core execution speed, we implement the 3D NMS and CAR operations as TensorFlow custom operations. Custom operations are C-coded algorithms compiled within TensorFlow sources and callable with the Python API of TensorFlow. In order to build these algorithms, we start from the two-dimensional C implementations available in TensorFlow sources, and we rewrite all functions to manage tensors with one more dimension. In particular, the resizing part of the 2D CAR operation proposes two classical interpolation methods: the nearest neighbor and the bilinear modes. We thus convert the nearest neighbor approach to 3D, and we implement the equivalent of the bilinear interpolation method at 3D, which is the trilinear interpolation method. We deliver safety tests for the 3D NMS and CAR. In order to allow an end-to-end back-propagation during training, we finally link to these operations their exact analytical gradients. The code of the custom operation is open source. We facilitate the portability of our work by adopting a containerized approach.

### 2.3 Experience datasets

We validate our Mask R-CNN implementation through two complementary experiences. The first one consists of training our network on a simple controlled environment, called the Toy dataset, whose purpose is to demonstrate that our 3D implementation is fully functional and to test the network limitations in the context of strong anisotropy and considerably weak signal-to-noise ratio. The use of such a synthetic dataset is essential due to the scarcity of 3D public datasets. The second validation illustrates the power of the 3D Mask R-CNN in a real use case, that is, the instance segmentation of PM embryo cells (Guignard et al., 2020b). Embryos show densely connected cells, with strong overlap between ground truth bounding boxes. For memory issues, we only use examples containing fewer than 300 cell instances. The COCO dataset, on which the 2D Mask R-CNN was benchmarked, exhibits roughly 4.5 instances per image on average, against 10 for the Toy dataset. With an average number of instances per image close to 125, the PM embryo dataset constitutes an extreme scenario compared to other datasets and allows us to test other limitations of this region-based method.

#### 2.3.1 Toy dataset

**FIGURE 1:**
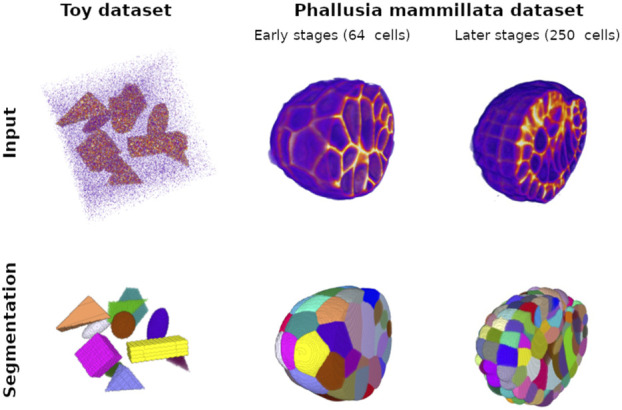
Ground truth training examples for the Toy dataset with cuboids, ellipsoids, and irregular pyramids and for the *Phallusia mammillata* dataset. The top pictures represent intensity maps of the input signal; strong intensity is displayed in orange. This signal corresponds to the objects in the case of the Toy dataset and the membrane signal in the SPIM fused images. Note that, in the case of the Toy dataset, we generate a special image with a signal-to-noise ratio much higher than the images used for training and validation for the sake of visualization (see implementation for creating original examples). Low intensity, mainly image noise, appears as purple. For display comfort, we eliminate low background values, and we crop the *Phallusia mammillata* images to exhibit the complexity of the internal structure of the membrane signal. The bottom pictures correspond to the respective instance segmentation of the top pictures: each color corresponds to the mask of one object instance in the input image. We show two developmental stages of the PM1 embryo: an early stage with a low number of cells and an intermediate stage with 250 cells.

As a controlled environment, the Toy dataset is automatically generated owing to well-mastered parameters such as image size, maximum instance number, or object shape. No data preparation or augmentation is hence performed. This dataset contains three object classes, in addition to the background class, allowing validation of all the 3D Mask R-CNN modules. For this paper, we generate 10,000 noised images of 128×128×128 voxels containing three types of objects: cuboids, ellipsoids, and irregular pyramids (see Figure 1). The minimal and maximal number of instances per image are 3 and 20, respectively, and the typical size of the objects goes from 15 voxels to 60 voxels. The aspect ratios between two axes of an instance range from 0.25 to 1.0, while the objects are generated with a randomly selected noise level, yielding instances that vary from clearly visible to nearly indiscernible objects that cannot be segmented using a simple Otsu method. The Toy dataset, therefore, allows rigorously challenging the 3D Mask R-CNN performance across a broad spectrum of shapes, anisotropies, and noise levels. The noised images come with their pair instance segmentations. We use 95% of the examples to train the 3D Mask R-CNN and the last 5% for validation. We use no test subset because the images created within this controlled environment all share the same distribution of signal and noise.

#### 2.3.2 PM dataset

The PM dataset consists of 1,742 3D isotropic images of 21 PM embryos acquired at regular time steps and various developmental stages (Guignard et al., 2020b). These images result from a multiview selective plane illumination microscopy (SPIM) acquisition and from a fusion method that merges the multiview anisotropic images into an isotropic one. The SPIM acquisition captures the embryo cell membranes labeled with a fluorophore, giving a raw representation of the embryo morphology. Any quantitative study of embryogenesis relies on such representations (see Figure 1). The remarkable transparency of PM embryos represents a rare opportunity in morphogenesis, allowing us to obtain excellent quality and resolution images. The PM dataset also offers high-quality cell instance segmentations. These ground truth segmentations are generated using ASTEC, a specialized algorithm that employs a seeded watershed approach. This algorithm accounts for the temporal propagation of the seeds, particularly focusing on cell division to optimize seed positions. The key strength of ASTEC is that the seeds for the initial time step are manually validated, ensuring the quality of the seed propagation. A data manual curation step is afterward performed using relevant tools (Leggio et al., 2019).

The PM images and segmentations are cropped and resized to the shape 256×256×256 voxels with conservation of the embryo aspect ratio. The 256-voxel shape is a good compromise between the conservation of the image details and processing speed for training and prediction. Ground truth cell bounding boxes and masks used for the Mask R-CNN training directly derive from the instance segmentations. As the number of training examples is low, we apply data augmentation to the 1,742 training examples. Using all possible rotations and flips, the augmentation factor rises to 48 and thus gives access to 83,616 examples.

We observe one major bias in the PM dataset: the image containing voluminous cells, which appear at the early stages of the morphogenesis, are under-represented among the cell instances in comparison to the images exhibiting tinier cells, which are found in all development stages of the embryo and especially in the later ones. This imbalance would lead the RPN and the Head modules to under-perform on these images. Therefore, we balance our data according to the volume of the cell bounding boxes.

We select 50% of the available examples in the augmented pool in order to quicken the training of the 3D Mask R-CNN while keeping 1% of this global set for validation. The training and validation subsets hence contain 21,740 and 220 examples, respectively. To evaluate the generalization of the training, we exclude from the training and validation sets the 100 raw examples of the PM1 embryo, which constitutes the most reliable series in the PM dataset as it is the most carefully curated series. We employ this test subset to evaluate the performance of the 3D Mask R-CNN on a completely original embryo development sequence.

### 2.4 Training procedure

Our objective is to develop a controlled training procedure that achieves satisfactory results as quickly as possible for both datasets. The fine-tuning of the 3D Mask R-CNN requires weeks or months. To significantly reduce the training time, we employ a two-step training strategy. We initially train the RPN for up to 20 epochs. Subsequently, we freeze the RPN and train the Head modules for 20 epochs. We generate the Head targets using 20% of the training set and train the Head only on this subset to decrease training time. In both scenarios, we select network hyperparameters that reduce training memory requirements and duration. For the Toy dataset, this optimization enables the training of the 3D Mask R-CNN within 2 days, including target generation for the Head. In the PM dataset case, we first optimize the hyperparameters using a lightweight version of the network to maximize detection and segmentation scores and then retrain with more RPN feature channels to enhance performance. Consequently, the network can be trained within 2 weeks for the latter case. We employ a batch size of one image, a stochastic gradient descent optimizer with a learning rate of 0.01, and an L2 regularizer for the total training loss.

Among the numerous hyperparameters of the 3D Mask R-CNN, we identify key parameters that significantly affect the network size, training duration, and performance. The two primary hyperparameters impacting memory usage are the number of channels of the RPN and the maximal number of RoIs handled by the Head. Our work is conducted on an IBM Power System AC922 computer equipped with an NVIDIA V100 graphic card featuring 32 GB of memory. This hardware configuration supports up to 256 RPN feature channels and 350 instances per input image. Concerning the network performance, alongside the detection of minimal confidence, the NMS threshold of the RPN influences the sorting of overlapping bounding boxes and the detection of connected objects within the input image. In addition, we observe that the ratio of positive to negative RoIs generated by the RPN during the Head training greatly affects the Head classifier performance and should be chosen carefully.

### 2.5 Validation metrics

We validate both experiments using precision, recall, and mean average precision (mAP) scores for an intersection over union (IoU) threshold defined as 50% , meaning that an instance is considered detected if it is correctly classified and it exhibits an IoU score over 50% with a ground truth instance. We also calculate the mean IoU of detected instances per image in order to measure the overall segmentation quality. These metrics are widely used and represent concrete information for non-specialists because the recall, which accounts for the false negative, can be interpreted as a detection score, and the precision, which gives insight into false positive prediction, can be interpreted as a reliability score.

## 3 Results

### 3.1 Toy dataset

The 3D Mask R-CNN trained over the Toy dataset shows fine results on the test subset with a median mAP of 99.1% and a mean IoU of 81.9% for matching ground truth and predicted objects. This relatively weak mean IoU performance arises from the important input noise. The median recall and precision scores are 100% and 92.9% , respectively. This high recall score indicates that the 3D Mask R-CNN successfully detects all instances in most test examples. However, the precision score suggests that the model predicts a significant number of false positives. Interestingly, we find that for both the misclassified and false negative detections, the aspect ratio distributions align with the ground truth distribution, indicating that the 3D Mask R-CNN handles anisotropy well. In contrast, the noise level emerges as the principal source of error, with the noisiest instances over-represented among the false negative and misclassified detections in regard to the ground truth distribution (see Supplementary Figures S1; Supplementary Figures S2). This outcome highlights the performance limitations of the 3D Mask R-CNN in the context of an unfavorable object signal-to-noise ratio.

### 3.2 PM dataset

#### 3.2.1 The 3D Mask R-CNN predicts high-quality cell instance segmentation in the PM dataset

**FIGURE 2:**
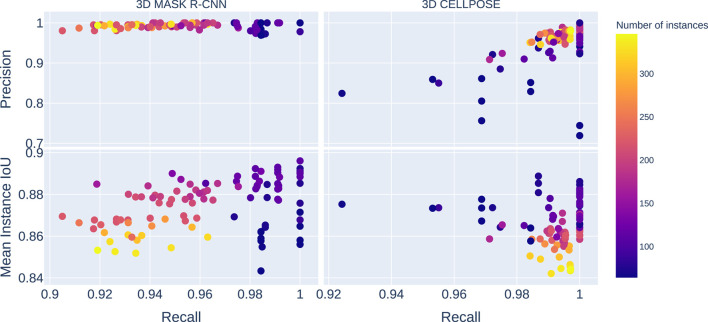
The plots on the left gather the 3D Mask R-CNN results, and the images on the right are the 3D Cellpose ones. The top plots represent the precision score against recall, while the bottom ones display the mean instance IoUs against recall. The color scale indicates the number of instances in each image in the test dataset. The *Y*-axis is shared between the horizontal pairs, and the *X*-axis is shared between vertical ones. The 3D Mask R-CNN shows better results than 3D Cellpose for precision and mean instance IoUs. The 3D Cellpose outperforms the 3D Mask R-CNN in the recall score case.

**FIGURE 3:**
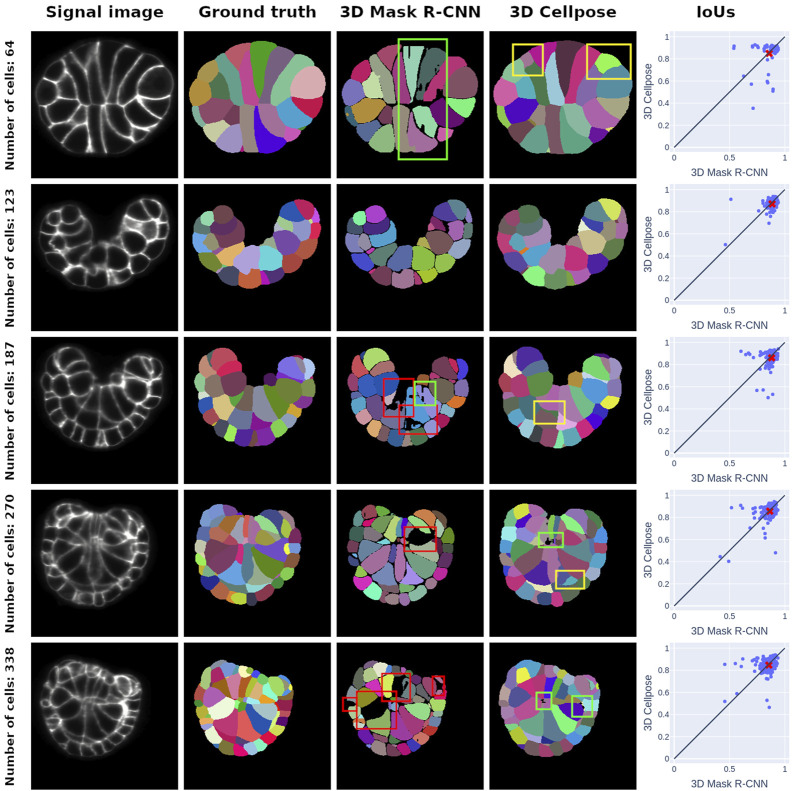
Middle-section plots of 3D PM1 signal images and segmentation predictions at various developmental stages. Each row concerns one signal image, whose stage is given by the ground truth cell number in the row title. The first column offers a representation of the SPIM images that we use as inputs for both networks, and the second column corresponds to ground truth ASTEC segmentations of the signal images. The third and fourth columns are the segmentations predicted by the 3D Mask R-CNN and the 3D Cellpose, respectively. The last column represents the cell IoUs for each prediction method in comparison with the ground truth masks. The mean of the distribution is given by a red cross. It consistently falls below the y=x line, indicating that 3D Mask R-CNN generally achieves a better IoU score on detected instances than 3D Cellpose. Some errors are shown with colored bounding boxes. The red boxes highlight detection errors or false negatives, the yellow boxes indicate the false positive detections, and the green boxes show the poor cell segmentations.

The results of the Mask R-CNN trained over the PM dataset (see Figure 2) show a good median recall score of 96.3% and excellent reliability with a median precision over 99.5% . The Mask R-CNN hence manages to detect almost all cells with an almost nonexistent number of false positives and also shows a low number of false negatives, which accounts for missing cells in the prediction. The recall deviation is wide, showing a value close to 98% for early development stage images and values down to 91% for later stages. The corresponding time steps indeed exhibit detection failures, which appear as holes within the embryo cell segmentation. The detection failures are blatant in the predicted segmentation images due to the region-based paradigm (see Figure 3). The mean average precision has a resulting median value of 95.8% .

The IoU score exhibits regular behavior close to its median value 87.7% , and a score approximately 86% for the later stages, which are good results, as the IoU is a score that strongly penalizes pixel misclassification. In order to analyze the segmentation errors, we display slices of some ground truth and predicted segmentation examples in Figure 3, where we observe the mask errors to be the most blatant. The detection errors penalizing the recall score are indicated with red bounding boxes. We can also identify poor-quality cell segmentations, marked with green boxes, especially for elongated cells, showing a plausible lack of training in the segmentation module. Another error source is the divergence between ground truth and predicted cell connectivity, which sometimes is significant, although these boundaries and cell connectivities appear to be well-conserved for most cases. By cell connectivity conservation, we refer to the ability of a segmentation method to reproduce the full connection of the embryo cells with no background voxels between instances.

#### 3.2.2 3D Mask R-CNN appears to be competitive against the state-of-the-art method

We compare the performance of the 3D Mask R-CNN with the state-of-the-art deep learning method for cell instance segmentation in microscopy images, 3D Cellpose, which is known to perform very well on a variety of microscopy styles as well as species (Stringer et al., 2020; Pachitariu and Stringer, 2022). In contrast with the 3D Mask R-CNN, 3D Cellpose does not rely on the region-based paradigm but on vector fields to delineate cells. 3D Cellpose consists of the application of the 2D Cellpose on all image slices, according to the three-space direction. The predicted 3D instance segmentation results from a fusion step of the three stacks. In the next paragraphs, we compare our predictions to those obtained using the 3D Cellpose pre-trained model called *cyto3* because it offers the best results not only in comparison to the other pre-trained model but also to a version of 3D Cellpose retrained over our data (see Supplementary Table S1).

3D Cellpose shows a better overall success than the 3D Mask R-CNN for cell detection (see Figure 2) with a median mAP value of 97.5% , owing to an excellent recall score whose median is 99.5% on the test subset. Interestingly, with a median precision score of 96.6% , 3D Cellpose is outperformed by the 3D Mask R-CNN. It appears that 3D Cellpose tends to predict many false positive cells (see Figure 3). The Cellpose mean IoU score has a median of 86.4% that is also less than the 3D Mask R-CNN score.

In Figure 3, we expose the typical segmentation errors of both methods by highlighting them with colored bounding boxes. 3D Cellpose also produces some poor segmentations (green boxes). 3D Cellpose mistakes are mainly false positives (yellow boxes). These errors, almost nonexistent in Mask R-CNN predictions, come from the vector field approach of 3D Cellpose, which can lead to confusion between signal noise and instances.

## 4 Discussion

### 4.1 Results illustrate the paradigm of each method

The high performance of the Mask R-CNN is directly linked to the region-based paradigm on which it relies, which ensures that the false positive detections remain low. The Mask R-CNN also shows a general under-segmentation tendency at instance boundaries, which comes from the mask processing at the RoI level. In consequence, the cell connectivity, while well conserved between correctly segmented cells, does not reach the esthetic of a watershed approach, at least at this point of training. In contrast, the 3D Cellpose approach offers a cell connectivity quality close to ASTEC. However, it predicts many false positive cells. The 3D Cellpose bias is thus to over-segment cells.

### 4.2 Use case evaluation

**FIGURE 4:**
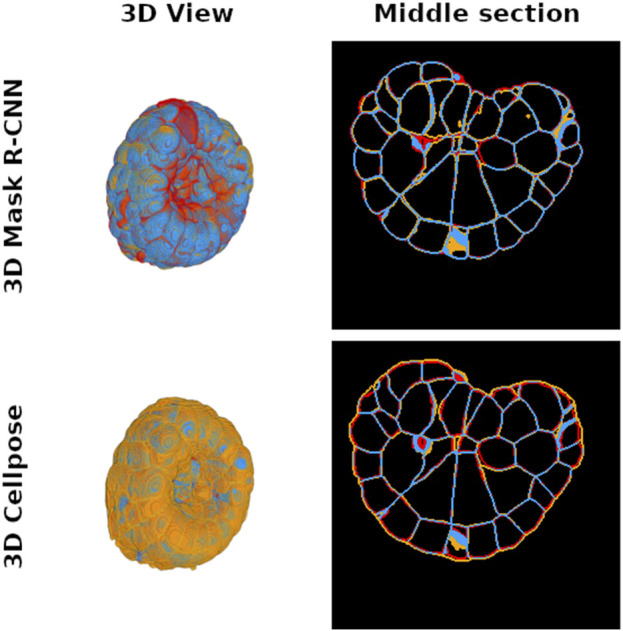
Distribution of true positive (blue), false positive (orange), and false negative (red) voxel maps after comparison between 3D Mask R-CNN (top pictures) and 3D Cellpose (bottom pictures) predictions and ground truth cell interface maps of a PM1 example. The left images show a 3D representation of each map to illustrate the global differences between the two tested methods, while an intermediate slice plot of the 3D interface maps is given in the right pictures. Each interface map is generated from the predicted instance segmentation by finding the boundaries of each instance label. The 3D Mask R-CNN conserves more cell interfaces than Cellpose.

Cell instance segmentation of a PM embryo is performed to measure cell instance volume or cell–cell contact surface. This information is important because it directly translates the coupling between genetic expression and morphogenesis regulation (Guignard et al., 2020b). Membrane conservation is thus a key criterion to validate the success of an instance segmentation deep learning method intended to ensure reliable experimental measurements in laboratory use. In order to evaluate the membrane conservation, we first generate the masks of the cell segmentation boundaries predicted by the 3D Mask R-CNN and 3D Cellpose, as well as the ground truth membrane masks given by ASTEC for the test subset examples. Afterward, we count the true positive voxels that correspond to the conserved part of the cell interface between ground truth and prediction (see Figure 4). We find that the average true positive voxel rate for the 3D Mask R-CNN is approximately 61.8% while the rate is 57.8% for 3D Cellpose. The 3D Mask R-CNN true positive rate rises to 63.7% if a simple one-voxel label expansion is applied to its predictions (see Supplementary Table S2 for detailed results). In addition, the proportion of false negatives is 2.1% for Cellpose and 1.2% for the Mask R-CNN ( 1.7% in case of label expansion). The false negative errors of the Mask R-CNN mainly come from bad segmentation and should decrease with longer training. These results indicate that the 3D Mask R-CNN predicts cell interfaces closer to the ASTEC gold standard than the interfaces inferred by 3D Cellpose. Training 3D Cellpose over our data does not change this result (see also Supplementary Table S2).

### 4.3 Perspectives

The 3D Mask R-CNN demonstrates its reliability and limitations on the Toy dataset and shows promising results for the instance segmentation of cell embryos and for morphogenetic quantitative studies. Its competitiveness against the state-of-the-art method is particularly evident in preserving important features such as instance interfaces. The analysis of segmentation errors reveals certain challenges, including fragmented cell segmentations and boundary divergence between instances due to under-training. Despite these issues, the 3D Mask R-CNN outperforms its competitor, 3D Cellpose, in terms of reproducing gold-standard cell interfaces. This finding highlights the Mask R-CNN as the most promising network for practical applications that require great precision.

The inference time of the 3D Mask R-CNN is relatively long, taking approximately 13 h for 100 time steps, whereas 3D Cellpose offers quicker predictions with a rough inference time of 3.5 h. This difference in speed illustrates the decision that bio-image analysts must make between a method like 3D Cellpose and a region-based approach like the 3D Mask R-CNN. Although 3D Cellpose may provide faster predictions, it may exhibit limited performance on specific usage. On the other hand, despite being slower in both training and inference, the 3D Mask R-CNN possesses the ability to reproduce the ground truth features. This trade-off between speed and accuracy should be carefully considered when choosing an appropriate method for specific bio-imaging analysis tasks.

## Data Availability

The datasets presented in this study can be found in online repositories. The names of the repository/repositories and accession number(s) can be found at: 3D Mask R-CNN data: https://figshare.com/articles/dataset/3D_Mask_R-CNN_data/26973085.
