# Supplementary material for: End-to-end 3D instance segmentation of synthetic data and embryo microscopy images with a 3D Mask R-CNN
Source: Front Bioinform. 2025 Jan 29;4:1497539. doi: 10.3389/fbinf.2024.1497539 (PMC11814465; doi:10.3389/fbinf.2024.1497539)
Supplement: Supplementary file 1 [file DataSheet1.pdf]

Supplementary Material

1 SUPPLEMENTARY TABLES AND FIGURES

1.1 Tables

Methods	mAP	Recall	Precision	Mean IoU
3D Mask R-CNN	95.8	96.3	99.5	87.7
3D Mask R-CNN (EL1)	96.0	96.3	99.6	87.9
3D Cellpose (*cyto3*)	97.5	99.5	96.6	86.4
3D Cellpose (retrained)	90.2	95.5	87.2	83.6

Table S1. Comparison of the instance segmentation methods that we investigate according to four metrics: the mean average precision (mAP), the recall and precision scores, and the mean Intersection over Union (IoU) of detected instances. The displayed results are the median of these metric distributions over the PM1 examples. The results of the 3D Mask R-CNN and 3D Cellpose (*cyto3*) are presented in the main article. The 3D Mask R-CNN (EL1) corresponds to the predictions of the 3D Mask R-CNN on which we apply a simple label expansion of one voxel, which is the simplest curation post-processing step that can be performed in order to improve the cell connectivity in the 3D Mask R-CNN segmentations. Such a curation allows to obtain slightly better results than the 3D Mask R-CNN. The 3D Cellpose (retrained) is a Cellpose version trained over a sample of the Phallusia mammillata train subset: we train it for 100 epochs over a sampling of $50,000$ 2D examples arising from the Phallusia mammillata train subset using *cyto2* pre-trained weights as a starting point. We did not include these results in the main article since the *cyto3* pre-trained model achieves a better performance.

Methods	TP rate	FP rate	FN rate	TN rate
3D Mask R-CNN	61.8	38.2	1.2	98.8
3D Mask R-CNN (EL1)	63.7	36.3	1.7	98.3
3D Cellpose (*cyto3*)	57.8	42.2	2.1	97.9
3D Cellpose (retrained)	57.8	42.2	2.3	97.7

Table S2. Comparison of the instance segmentation methods that we investigate according to four metrics for the cell interface analysis: the true positive (TP) voxel rate, the false positive (FP), the false negative (FN) and the true negative (TN). The displayed results are the average of these metric distributions over the PM1 examples. The analysis in Table S1 also stands for these results.

1.2 Figures

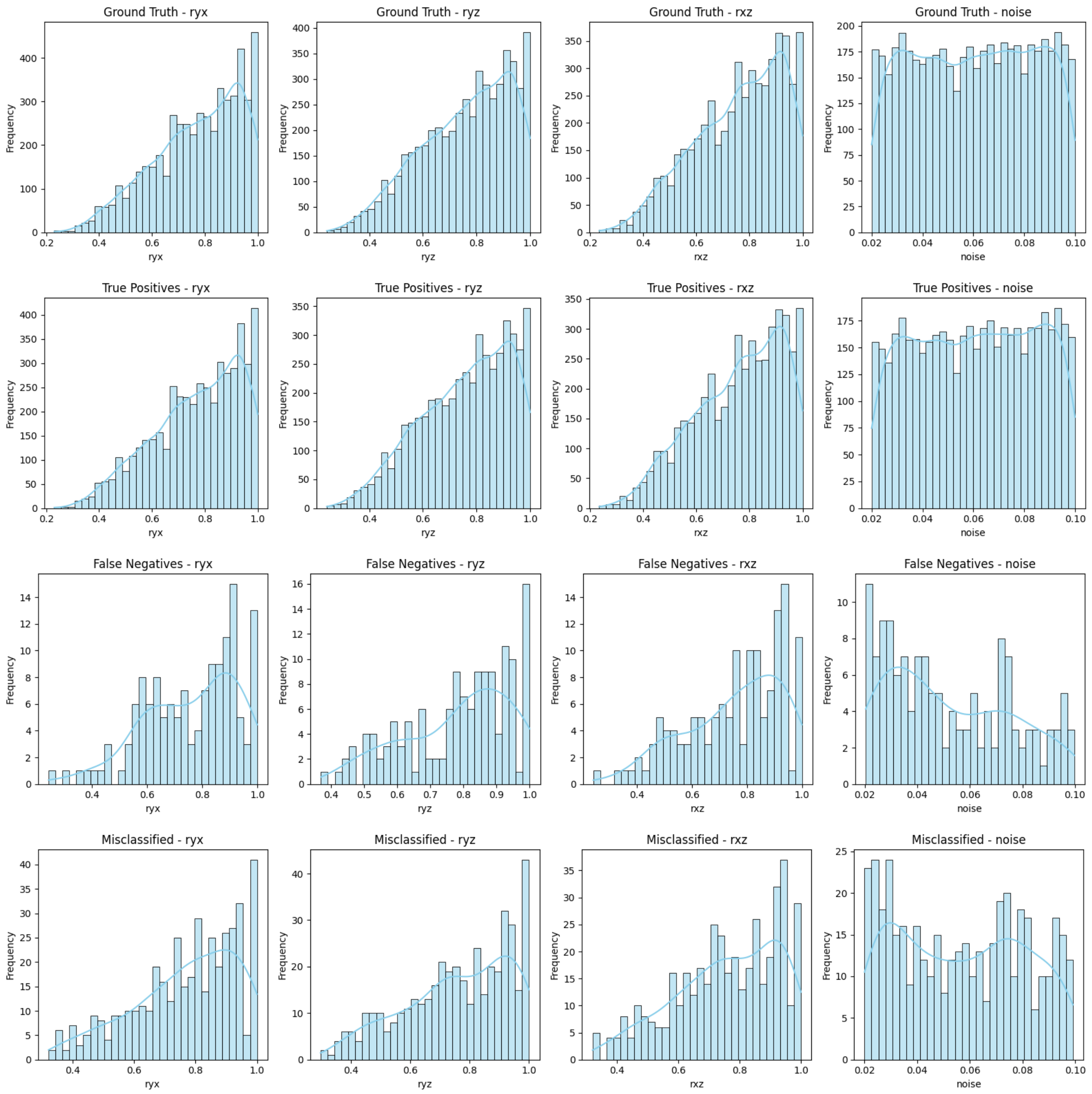

Figure S1. The image presents a grid of histograms displaying the distribution of instances in the Toy dataset test subset according to four metrics, one for each column: the three object ratio of the 3D objects (precisely, the aspect ratio of their bounding boxes) and their noise level. For instance, the aspect ratio between the object x-length and y-length is simply noted *ryx*. The noise level gives the intensity associated to the object during the generation: the lower the noise level, the weaker the object signal. The noisiest object are associated with the lowest noise level. The rows represent different data categories. The first row corresponds to the ground truth data, indicating the raw distribution of aspect ratios and noise levels in the test subset. The second row shows the distributions of the true positives, that are the objects being correctly detected and classified during inference by the 3D Mask R-CNN. These plots demonstrate that most instances are detected by the 3D Mask R-CNN. The third and the fourth rows display the distributions of the false negatives (missing detection) and misclassified predictions respectively. Interestingly, the aspect ratio distributions for these two last categories align with the ground truth distributions while the noise level one exhibit a bias in favor of the lowest noise level. See Figure S2 for a complementary analysis. We represent with an overlay the density curves of the distributions for better visualization of the trends.

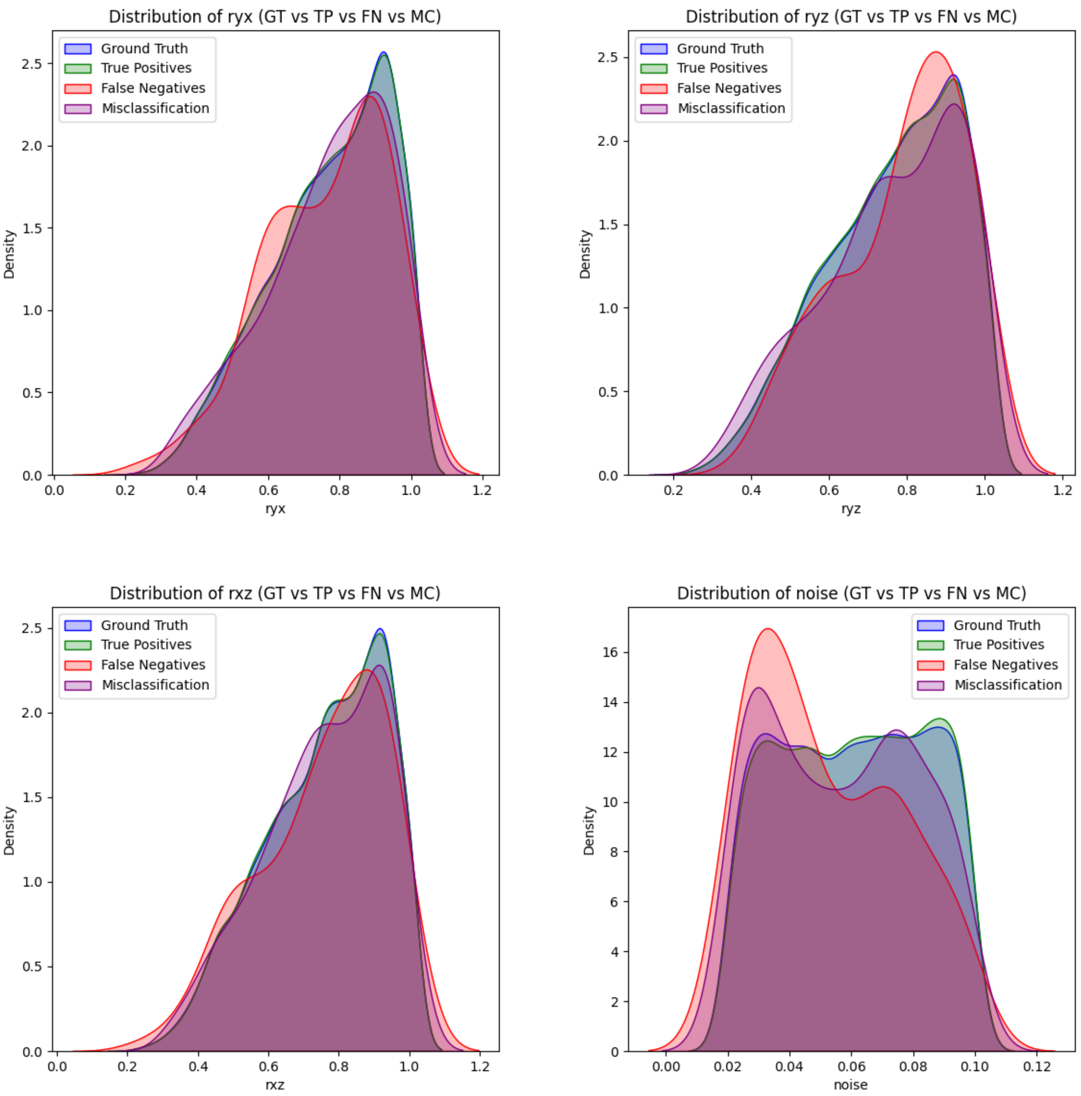

Figure S2. The image displays a set of four density plots, each illustrating the correlation distributions for different metrics: the three aspect ratios *ryx*, *ryz*, *rxz*, and noise level. The purpose of these plots is to compare the distribution of four categories across each metrics: ground truth (blue curve), true positives (green curve), false negatives (red curve) and misclassifications (purple curve). We observe that the *ryx*, *ryz* and *rxz* aspect ratios distributions roughly align with the ground truth one, indicating that a range of aspect ratio values does not affect the prediction success of the 3D Mask R-CNN. However, the noise level density plot (bottom right) shows a bias in favor of the lowest noise levels that correspond to the object with the less signal intensity. Such objects introduce false negative and misclassification errors during inference.

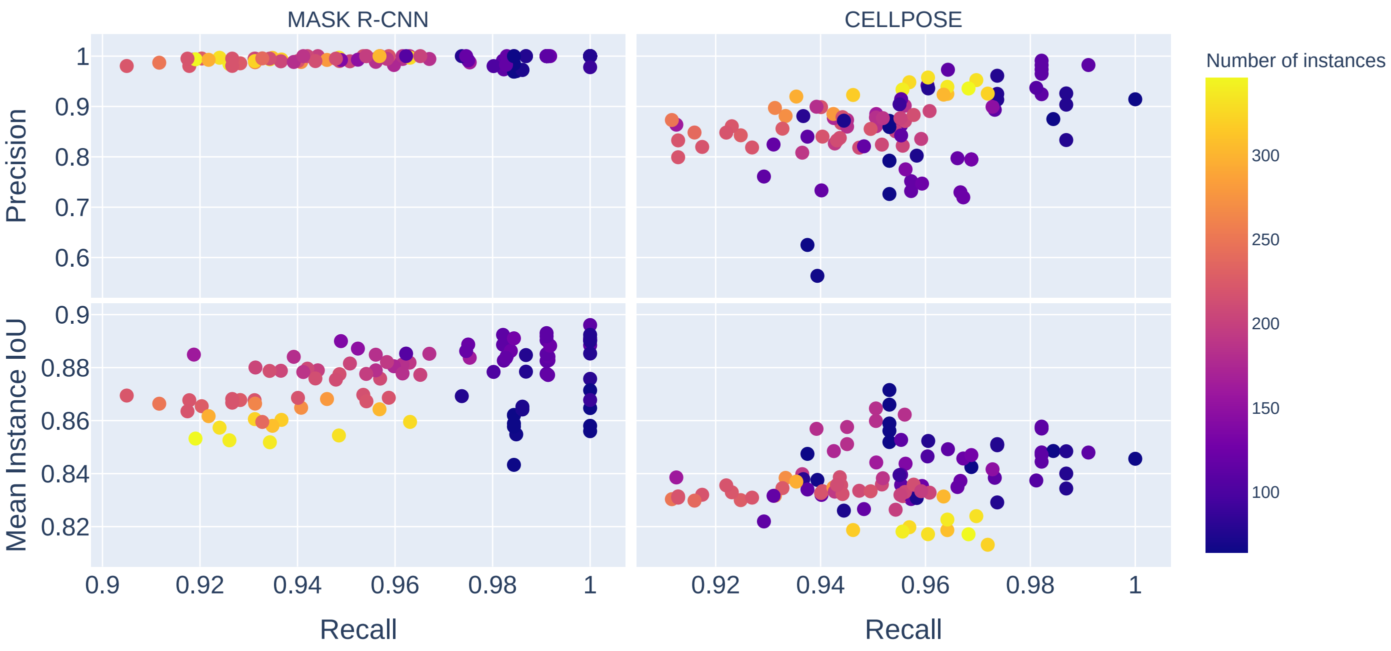

Figure S3. Left plots gather the 3D Mask R-CNN results, and the right pictures the 3D Cellpose ones. The Cellpose model used to obtain these results is the Cellpose retrained over our data (see Table S1 for training details). Top plots represent the precision score against recall, while the bottom ones display the mean instance IoUs against recall. The color scale indicates the number of instances in each image in the test dataset. Y-axis are shared between the horizontal pairs and X-axis are shared between vertical ones. The 3D Mask R-CNN shows better results than retrained 3D Cellpose.
